# White Matter Integrity and Treatment-Based Change in Speech Performance in Minimally Verbal Children with Autism Spectrum Disorder

**DOI:** 10.3389/fnhum.2017.00175

**Published:** 2017-04-05

**Authors:** Karen Chenausky, Julius Kernbach, Andrea Norton, Gottfried Schlaug

**Affiliations:** ^1^Department of Neurology, Music, Neuroimaging, and Stroke Recovery Laboratory, Beth Israel Deaconess Medical CenterBoston, MA, USA; ^2^Department of Neurology, Harvard Medical SchoolBoston, MA, USA; ^3^Department of Nuclear Medicine, University Hospital, RWTH Aachen UniversityAachen, Germany

**Keywords:** autism, speech therapy, intonation, AMMT, minimally verbal, speech development, speech errors, white matter integrity

## Abstract

We investigated the relationship between imaging variables for two language/speech-motor tracts and speech fluency variables in 10 minimally verbal (MV) children with autism. Specifically, we tested whether measures of white matter integrity—fractional anisotropy (FA) of the arcuate fasciculus (AF) and frontal aslant tract (FAT)—were related to change in percent syllable-initial consonants correct, percent items responded to, and percent syllable insertion errors (from best baseline to post 25 treatment sessions). Twenty-three MV children with autism spectrum disorder (ASD) received Auditory-Motor Mapping Training (AMMT), an intonation-based treatment to improve fluency in spoken output, and we report on seven who received a matched control treatment. Ten of the AMMT participants were able to undergo a magnetic resonance imaging study at baseline; their performance on baseline speech production measures is compared to that of the other two groups. No baseline differences were found between groups. A canonical correlation analysis (CCA) relating FA values for left- and right-hemisphere AF and FAT to speech production measures showed that FA of the left AF and right FAT were the largest contributors to the synthetic independent imaging-related variable. Change in percent syllable-initial consonants correct and percent syllable-insertion errors were the largest contributors to the synthetic dependent fluency-related variable. Regression analyses showed that FA values in left AF significantly predicted change in percent syllable-initial consonants correct, no FA variables significantly predicted change in percent items responded to, and FA of right FAT significantly predicted change in percent syllable-insertion errors. Results are consistent with previously identified roles for the AF in mediating bidirectional mapping between articulation and acoustics, and the FAT in its relationship to speech initiation and fluency. They further suggest a division of labor between the hemispheres, implicating the left hemisphere in accuracy of speech production and the right hemisphere in fluency in this population. Changes in response rate are interpreted as stemming from factors other than the integrity of these two fiber tracts. This study is the first to document the existence of a subgroup of MV children who experience increases in syllable- insertion errors as their speech develops in response to therapy.

## Introduction

The current diagnostic criteria for autism spectrum disorder (ASD) include deficits in social communication and the presence of repetitive behaviors or restricted interests (American Psychiatric Association and Task Force on DSM-5, 2013). ASD is usually diagnosable by the age of 3 years, but ~25%–46% of children diagnosed with ASD remain minimally verbal (MV) past 5 years of age (Tager-Flusberg et al., 2005; Kasari et al., 2013; Tager-Flusberg and Kasari, 2013; Rose et al., 2016). Several studies have shown that, in MV children and adults with ASD, communication impairment is an independent predictor of high rates of aggression (Hartley et al., 2008; Matson and Rivet, 2008), that self-injurious behavior is negatively related to expressive language level (Baghdadli et al., 2003; Dominick et al., 2007), and that problem behavior decreases when children learn effective and appropriate communication skills (Buschbacher and Fox, 2003). Thus, communication therapy is critical for MV children with ASD. The position statement of the American Speech-Language-Hearing Association on service delivery for persons with mental retardation or developmental disabilities states that these individuals “have a basic right to influence their life circumstances and opportunities through communication” (American Speech-Language-Hearing Association, 2005). Nonetheless, the task is daunting; well-described, efficacious therapies are rare; and results are limited. All of these factors underscore the need for continued research to understand why these children remain MV and which therapies are most appropriate for which individuals.

Becoming a competent user of spoken language requires several types of skills. Among them are: (a) the intent to communicate; (b) lexical, semantic and syntactic knowledge; (c) the ability to imitate; and (d) motor planning for production of intelligible speech. Thus, the spoken modality may not be appropriate for all MV children with ASD. Previous spoken language therapies for MV children with ASD have focused mainly on the first two skills (Prizant et al., 2000; Rogers et al., 2000; Goldstein, 2002; Paul, 2008; Paul et al., 2013). However, some types of therapy also target the latter two (Chumpelik, 1984; Koegel et al., 1998; Rogers et al., 2006; Pickett et al., 2009; Paul et al., 2013).

Recently, interventions that involve intoned (sung) rather than spoken stimuli have been shown to be effective in improving social responses and speech output in children with MV ASD. For example, Paul et al. (2015) showed that singing directives, rather than speaking them, resulted in a greater response rate to name and to social gestures in three MV children with ASD. However, Lim and Draper (2011) showed that sung instructions were as effective as spoken instructions in eliciting four types of verbal communication in 22 verbal and preverbal preschool children with ASD.

In terms of speech production, Wan et al. (2011) showed that Auditory-Motor Mapping Training (AMMT), an intervention involving repetition of intoned rather than spoken stimuli, significantly improved speech fluency in six children with MV ASD between the ages of 5 and 9, and resulted in an average improvement of 19.1% syllables approximately correct after 40 treatment sessions. Building on this work, Chenausky et al. (2016) compared AMMT to a non-intonation-based control intervention, Speech Repetition Therapy (SRT), in 23 children receiving AMMT and seven receiving SRT. After 25 sessions of treatment, the AMMT group improved by 19.4% syllables approximately correct, significantly higher than the SRT group’s improvement of 3.6%.

In addition to improvement in the outcome measures discussed above, both Wan et al. (2011) and Chenausky et al. (2016) noted other positive changes in the AMMT participants after treatment, such as increased ability to vocalize and an improvement in the range and complexity of their speech output. However, a wide range of improvement in speech fluency was found among the children in the two studies. Specifically, in Chenausky et al. (2016), while 19 of the 23 AMMT participants showed a statistically significant improvement in number of syllables approximately correct per stimulus after treatment, the remaining four did not experience such an improvement. In addition, some children began producing a high rate of extra syllables in addition to the two in the stimuli. These findings motivated the current study, which includes 10 children from Chenausky et al. (2016) who were able to participate in an MRI study at baseline.

Finding that an intonation-based therapy is capable of producing greater improvement in speech fluency than a non-intonation-based therapy is an important first step in treatment research for this population. However, it is still unknown how AMMT works and why it works for some, but not all, children with MV ASD. We also know little about what neural pathways are related to the MV phenotype in ASD and whether or not the integrity of these pathways predicts spoken-language therapy success. Being able to predict which children may benefit from AMMT (or any other therapy) will improve clinical decision-making, as it will enable us to select treatments that have a high probability of success for individual children, saving precious developmental time and maximizing treatment outcomes. In addition, the information we gain from investigating the neural structure for language in these children advances our knowledge of the neural control of speech in both children with ASD and typically developing children. The current study is a preliminary investigation of the neural predictors of response to AMMT treatment in children with MV ASD.

Two growing bodies of research from the past half-decade have shed some light on the possible neural basis for the spoken language deficits observed in children with MV ASD (Broce et al., 2015). One of them has implicated the arcuate fasciculus (AF) in the challenges these children face in acquiring spoken language. The AF is a fiber tract connecting primary and higher order auditory-perceptual regions in the temporal lobe directly to motor, pre-motor and motor planning regions in the posterior inferior portion of the frontal lobe; and is considered to form part of the dorsal language pathway (Friederici, 2009) of Hickok and Poeppel’s dual-stream model (Hickok and Poeppel, 2004). The AF is composed of three segments (Catani et al., 2002, 2007). For example, in the left hemisphere, the anterior indirect segment connects the posterior inferior frontal cortex (Broca’s area) with the inferior parietal cortex (Geschwind’s area), and the posterior indirect segment connects Geschwind’s area and superior posterior inferior frontal cortex (Wernicke’s area). Finally, the direct segment connects Broca’s and Wernicke’s areas to each other. All connections are bidirectional, providing the backbone for the feedforward and feedback control of auditory-motor functions. The AF has been linked in adults to skills such as mapping auditory stimuli to articulatory movements (Saur et al., 2008; Maldonado et al., 2011) and to the ability to use phrase-structure syntax (Friederici et al., 2006; Wilson et al., 2011). For example, radial diffusivity of the AF’s left direct segment has been found to be inversely correlated with word learning performance (López-Barroso et al., 2013). Catani et al. (2005) have linked the direct segment of the AF with the ability to repeat speech and the anterior and posterior indirect segments with the ability to produce spontaneous speech.

The other body of research concerns the frontal aslant tract (FAT), which links the posterior inferior region of the frontal lobe with the frontomesial region of the brain, most likely including the cingulate motor regions and the supplementary/pre-supplementary motor areas (Catani et al., 2013). In adults, integrity of the FAT has been shown to be positively correlated with fluency and speech initiation in patients with primary progressive aphasia (Catani et al., 2013; Kemerdere et al., 2016), in patients with focal lesions involving the FAT (Vassal et al., 2014; Kinoshita et al., 2015), and in adults with developmental stuttering (Kronfeld-Duenias et al., 2016).

One final aspect of the previous research examining the structure and function of the AF and FAT is important for the current study: that of laterality. Though language functions have long been considered to be left-lateralized, Catani et al. (2007) provided evidence that: (a) lateralization patterns for language networks vary in healthy adults; and (b) better performance on a word-learning task was associated with bilateral representation of the direct segment of the AF. Similarly, López-Barroso et al. (2013) found that word-learning performance was negatively correlated with lateralization indices of the direct segment of the AF. Lai et al. (2012) showed that children with ASD had both lower fractional anisotropy (FA) of the left dorsal language pathway and lower functional response to speech in the left inferior frontal gyrus than typically developing controls, and that these two variables were correlated in participants with ASD. However, for children with ASD, activation in the inferior frontal gyrus was higher for song and lower for speech during a listening task than in controls. Finally, and most relevantly for the current study, Wan et al. (2012) found that four of five MV children with ASD showed reversed volume asymmetry for the AF, whereas five of five typical controls showed the expected leftward asymmetry.

Less has been written about lateralization of the FAT, but there are a few studies that touch on the topic. Meyer et al. (2002) demonstrated that, in typical adults, fronto-opercular regions were activated more in the right than in the left hemisphere while listening to stimuli consisting of speech melody (prosodic or intonational information) alone. Broce et al. (2015) found evidence of right laterality of the FAT in typically developing children aged 5–8 years old, opposite to the pattern found in right-handed adults. Kinoshita et al. (2015) showed that subcortical electrostimulation of the left FAT in adults inhibited speech, but stimulation of the right FAT induced no speech disturbances. Finally, Sharda et al. (2016) showed that while sung words activated temporal regions bilaterally in both children with ASD and neurotypical controls, spoken-word perception was more right-lateralized in children with ASD. In addition, participants with higher verbal ability showed greater structural covariance between the left inferior frontal gyrus and right frontal regions, regardless of diagnosis. Thus, there is evidence that language is processed bilaterally in AF and FAT, both in typical speakers and in children with ASD.

In this study, we wanted to investigate the relationship of change in speech production variables after AMMT to fiber tract integrity of the AF and the FAT, as measured by FA, with the goal of being able to predict which children would be most likely to benefit from AMMT. Because of their sensitivity in measuring connectivity in the brain, FA values have become one of the most frequently used diffusivity-derived parameters in various research studies. In several of our previous studies in adults (e.g., Rüber et al., 2015), we were able to show that between-group differences in tract-FA values correspond to behavioral differences, and within-group changes in tract-FA and voxel-by-voxel FA values correlate with behavioral changes after intensive, long-term treatment (Wan et al., 2014), thus confirming FA values’ sensitivity as a measure of between-group differences and within-group changes in behavioral outcomes. Here, we used FA values for the AF and FAT pathways bilaterally to assess their relationship to changes in speech fluency in MV children with ASD after 25 sessions of AMMT. This led to the following research questions and hypotheses.

1. How are changes in measures of speech fluency related to integrity of the AF and the FAT in children with MV ASD? We hypothesized that the integrity of the AF would be most significantly related to speech production accuracy, and integrity of the FAT would be most significantly related to effortfulness of speech production.
2. Which hemisphere do children with MV ASD rely on for speech production? Because brain lateralization for language differs in our participants compared to typically-developing controls, and because intonation is typically processed in the right hemisphere, we hypothesized that integrity of the right-hemisphere AF and FAT would be more closely related to improvement in speech fluency in these children.

## Materials and Methods

### Participants

Participants in this study included 23 children with MV ASD (20 male), between the ages (year;months) of 3;5 and 9;8, who participated in at least 25 sessions of AMMT; other outcomes from this participant group were reported in Chenausky et al. (2016). Ten of the 23 children (eight male, mean age 6;10 ± 1;4 SD), had successful MR imaging studies at baseline. The children who participated in imaging studies are compared on a variety of behavioral measures to the remaining 13 AMMT participants (12 male, mean age 6;4 ± 1;10 SD). Because all of these children were participants in pilot phases of AMMT designed to demonstrate feasibility and effectiveness of the treatment (described in more detail in Wan et al., 2011; Chenausky et al., 2016), a randomized controlled design was not employed. Table 1 details the characteristics of the 10 participants who had successful MRI studies and the 13 participants who were not imaged. An additional seven MV children with ASD (five male, mean age 5;7 ± 1;5 SD) who received 25 sessions of SRT, a non-intonation based control treatment, are reported here. One-way ANOVAs were performed on all baseline measures, with group as a between-subjects factor, to identify any between-group differences. No ANOVAs were significant, indicating no between-group differences on Baseline measures.

**Table 1:** **Participant characteristics at baseline**.

			Age (year; month)	CARS^1^ score	Baseline phonemic inventory^2^	FM^3^	VR^4^	RL^5^	EL^6^
AMMT *n* = 23	Imaged *n* = 10	Mean ± SD	6;10 ± 1;4	38.8 ± 5.9	7.4 ± 4.8	30.2 ± 10.8	32.8 ± 10.0	23.0 ± 5.3	11.6 ± 0.9
		Range [min, max]	[5;4, 8;11]	[30.0, 47.0]	[3, 18]	[19, 45]	[20, 48]	[15, 29]	[11, 13]
	Non-imaged *n* = 13	Mean ± SD	6;4 ± 1;10	36.5 ± 5.7	7.5 ± 4.6	22.0 ± 2.4	23.4 ± 6.0	17.9 ± 7.3	10.6 ± 2.2
		Range [min, max]	[3;5, 9;8]	[30.0, 47.0]	[2, 16]	[18, 26]	[13, 31]	[10, 31]	[8, 14]
SRT *n* = 7		Mean ± SD	5;7 ± 1;5	36.6 ± 2.6	8.9 ± 5.9	28.1 ± 10.8	29.3 ± 12.1	18.7 ± 11.3	11.7 ± 3.9
		Range [min, max]	[3;10, 8;5]	[33.0, 40.0]	[2, 14]	[20, 47]	[17, 46]	[8, 36]	[6, 19]

*^1^CARS: Childhood Autism Rating Scale (40) score > 30 to confirm autism spectrum disorder (ASD) diagnosis. ^2^The phonemic inventory is the number of English vowels and consonants a child was able to imitate (max = 31 phonemes). ^3^FM: Fine Motor subscale of the Mullen Scales of Early Development (MSEL). Raw scores are used for all MSEL subscales; T-scores are generally uninformative for this population. Gross Motor scores are not listed, as this scale is only normed up until age 2;10. ^4^VR: Visual Reception subscale of the MSEL. ^5^RL: Receptive Language subscale of the MSEL. ^6^EL: Expressive Language subscale of the MSEL*.

Children were recruited from multiple autism clinics and resource centers serving the Greater Boston area. The study was approved by the Institutional Review Board of Beth Israel Deaconess Medical Center with written informed consent from the parents of all participants. The parents of all children gave written informed consent prior to enrollment, in accordance with the Declaration of Helsinki. The protocol was approved by the Institutional Review Board of Beth Israel Deaconess Medical Center. All procedures were conducted according to the approved protocol.

Diagnostic status was confirmed by a Childhood Autism Rating Scale (CARS; Schopler et al., 1988) score greater than 30. MV status was determined by parental report of expressive vocabulary less than 20 words and confirmed by clinical observation that the child produced fewer than 20 spontaneous (non-echoed) words and no word combinations during baseline assessment. For reference, a common figure used as a developmental milestone is an expressive vocabulary of at least 50 words and the use of some word combinations by age 24 months (Rescorla, 1989). Inclusion criteria were the ability to correctly repeat at least two English speech sounds, participate in table-top activities for at least 15 min at a time, follow one-step commands, and imitate simple gross motor and oral motor movements such as clapping hands and opening mouth.

Exclusion criteria included the presence of major neurological conditions (e.g., tuberous sclerosis), moderate to severe motor disabilities (e.g., cerebral palsy), sensory disabilities (e.g., hearing or vision impairment), or genetic disorders (e.g., Down Syndrome) that could potentially explain their MV state. While in the study, participants continued with their regular school programs but did not enroll in any other new treatments or speech therapy activities outside of school.

### Study Design

#### Behavioral Assessments

All participants underwent at least three baseline assessments prior to treatment and a probe assessment after 25 therapy sessions. Multiple baseline assessment sessions were administered to each child in order to establish a stable level of pre-therapy performance and to give children time to acclimate to the space and personnel. Change scores were calculated by subtracting each child’s best Baseline performance for the three speech production measures examined from their performance during the post-25 (P25) probe session. A child’s best Baseline was the assessment during which he or she produced the highest number of syllables approximately correct, as described in Chenausky et al. (2016).

##### Stimuli

A set of 30 high-frequency bisyllabic words/phrases pertaining to common objects (“bubbles”), actions (“all done”) or people (“mommy”) relevant to children’s activities of daily living were used in this trial. Twenty-eight of the stimulus words are included in the MacArthur-Bates Communication Development Inventory (Fenson et al., 1993). The remaining words are among the top 1008 most frequently used words from the Corpus of Contemporary American English (Davies, 2008). The same stimuli were used for both AMMT and SRT treatment sessions and assessments.

##### Treatment

Of the participants reported here, 23 received AMMT, an intonation-based treatment designed to increase spoken language fluency, which is described in detail in Wan et al. (2011) and Chenausky et al. (2016). Ten of these children also participated in an imaging study at Baseline; the remaining 13 did not. An additional seven participants received SRT, a non-intonation-based control treatment. All participants received at least 25 daily treatment sessions of therapy, each approximately 45 min in length. Since children in different phases of the study received different numbers of sessions, children’s best Baseline performance is compared to their performance after 25 treatment sessions.

For AMMT sessions, stimuli were intoned (sung) on two pitches, one per syllable, while the therapist and child simultaneously tapped electronic drums tuned to the same two pitches that were being sung. At each session, children were given five opportunities per stimulus to respond, either singing in unison with the therapist, repeating after the therapist, or completing a cloze (fill-in-the-blank) task. SRT sessions were structured in the same manner as AMMT sessions, but stimuli were spoken, not intoned; and neither drums nor bimanual tapping were used.

##### Measures of speech production

Three measures were used to assess changes in speech fluency. First, to track participants’ emerging ability to correctly produce the stimuli, a version of Percent Consonants Correct (Shriberg and Kwiatkowski, 1982) was employed. Percent Consonants Correct was developed to quantify the severity of developmental speech-sound disorders and was intended for use on connected, spontaneous speech samples. However, given the paucity of spontaneous vocalizations of any type in MV children, we modified the measure to apply to attempts at imitation of the AMMT stimuli. Since the rate of correct production of consonants in clusters or in syllable-final position was essentially zero in our participants, only syllable-initial singleton consonants were included in the analysis. Of the 60 syllables present in our set of 30 bisyllabic stimuli, a total of six syllables began with either no consonant or a consonant cluster and were thus excluded from this analysis, leaving a total of 54 consonant-vowel (CV) syllables to analyze. % Syllable-Initial Consonants Correct was calculated over these CV syllables by dividing the number of syllable-initial consonants a child produced that matched the target by the total number of syllable-initial consonants. For example, if a child said “dodo” for “doggie”, % Syllable-Initial Consonants Correct was 50% (one of two initial consonants in “doggie” was produced correctly).

Next, we used a measure designed to capture ease of speech production. Most children in the study did not respond to all prompts, but they generally increased their response rate over the course of therapy. To capture this change, we assessed the proportion of prompts to which they did respond. The total number of prompts per session is 150 (5 opportunities to respond per stimulus × 30 stimuli). Vocalizations such as crying/fussing, screaming/squealing, moaning, or laughing were not counted as attempts at imitation. % Responses was the percentage of prompts per session for which children attempted to imitate the target.

Our final measure was designed to capture effortfulness of speech production. Because some children produced more than two syllables per stimulus item, we also examined the rate of extra syllable production. A syllable insertion was scored if the child produced more than two syllables in response to a two-syllable prompt. For example, if a child said “buh buh uh” or “uh buh buh” for “bubble”, “uh” counted as one syllable insertion. The production “uh buh buh uh” counted as two syllable insertions, as did “uh uh buh buh uh uh” and so on. Syllables inserted between the two syllables of the stimulus were counted only once. % Syllable Insertions was the proportion of CV syllables with syllable insertion errors. Table 2 shows performance data from the 10 imaged AMMT participants in the current study, as well as data from the 13 comparison AMMT participants and the seven SRT participants.

**Table 2:** **Change score summary statistics**.

			Change in % Syllable-Initial consonants correct	Change in % Responses	Change in % Syllable Insertions
AMMT *n* = 23	Imaged *n* = 10	Mean ± SD	10.8 ± 16.7	18.1 ± 24.9	3.3 ± 6.6
		Range [min, max]	[−12.6, 35.8]	[−16.1, 78.3]	[−4.7, 15.2]
	Non-Imaged *n* = 13	Mean ± SD	16.0 ± 11.5	20.3 ± 25.4	13.9 ± 15.4
		Range [min, max]	[2.3, 35.2]	[−49.4, 49.4]	[−0.8, 49.6]
SRT *n* = 7		Mean ± SD	0.7 ± 10.0	17.7 ± 18.0	−4.9 ± 11.9
		Range [min, max]	[−11.1, 18.4]	[−12.2, 34.4]	[−29.8, 6.4]

As is clear from Tables 1, 2, the participants in this study displayed a range of abilities. For example, Table 1 reports raw scores for each group on the four subscales of the Mullen Scales of Early Learning (MSEL; Mullen, 1995) that are normed for children in this age range. Raw scores are reported because T-scores (standard scores with a mean of 50 and standard deviation of 10) are generally uninformative for this population, given the degree of delay in all domains that these children experience. MSEL scores are available for 5 of the 10 imaged AMMT, 7 of the 13 non-imaged AMMT participants, and for seven of seven SRT participants (i.e., for participants in the second and later phases of the work). There were no significant differences on MSEL score at Baseline between the imaged AMMT and non-imaged AMMT participants, or between the imaged AMMT and the SRT participants, as determined by two-tailed *t*-tests. In terms of change scores, listed in Table 2, again there were no significant differences between the imaged AMMT group and either the non-imaged AMMT group or the SRT group (again using two-tailed *t*-tests). However, the overall AMMT group did experience significantly greater improvement on % Syllable-Initial Consonants Correct, as established by an independent-samples *t*-test on the change score (AMMT mean 12.6% improvement, SD 15.0; SRT mean 0.7%, SD 10.0, *p* = 0.03). This between-group difference shows that the improvements are due to treatment, and not to just the passage of time.

##### Transcription

All Baseline and probe sessions were videotaped for offline transcription of children’s responses to the prompts. Inter-rater transcription reliability for consonants was 70.1% and associated with a Cohen’s *κ* = 0.547, *p* < 0.0005. This percent agreement rate is comparable to previously published figures on infant babbles of 76.8% for consonants (Davis and MacNeilage, 1995).

#### Image Acquisition

To maximize children’s ability to participate in the imaging study without sedation and to minimize movement, they were imaged at night while asleep. For all 10 imaged subjects, anatomical T1-weighted images with a resolution of 1.0 mm^3^ (FOV: 25.6 cm, echo time [TE] 3.39 ms, repetition time [TR] 2530 ms, flip angle 9 degrees) and diffusion tensor images (TE = 78 ms, TR = 11.5 s, flip angle 90°, FOV = 198 mm, slice thickness of 2 mm; resolution of 1.72 × 1.72 × 2 mm, obtaining 65 diffusion directions with a *b*-value of 3000 s/mm^2^, one image with *b* = 0 s/mm^2^) were acquired on a Siemens Trio Tim scanner.

##### DTI preprocessing

All diffusion-weighted images were processed and analyzed using the FDT (FMRIB’s Diffusion Toolbox) as part of the FSL software library^1^. We used an affine registration to the first volume to correct for eddy currents and head motion. All non-brain voxels were deleted using the Brain Extraction Tool (Smith, 2002) with a fractional intensity threshold of 0.2 to ascertain that all brain voxels were still included. All binary masks were then inspected and, if necessary, modified by hand to ensure accuracy. Next, a diffusion tensor model was fitted at each voxel using DTIFIT generating FA images. All FA images were normalized to the FMRIB standard FA template using the linear and non-linear image registration tool (Jenkinson and Smith, 2001; Greve and Fischl, 2009).

##### Tractography

We generated canonical templates of the AF and FAT from four typically developing children and 12 healthy adult subjects. The canonical tract templates derived from the children showed more variability, mainly because of the limited number of subjects being used to generate these canonical tracts. Since the adult templates were based on more subjects and showed less variability, they were used to extract FA and other diffusivity values. We verified that the extents and anatomical relationships of the FAT and the AF canonical tracts were similar between the child and adult templates. We did not specifically compare different segments of the AF or the FAT between typically developing children and adults. However, we found the canonical tracts of adults to be an accurate representation of the tracts found in neurotypical children.

**Figure 1:**
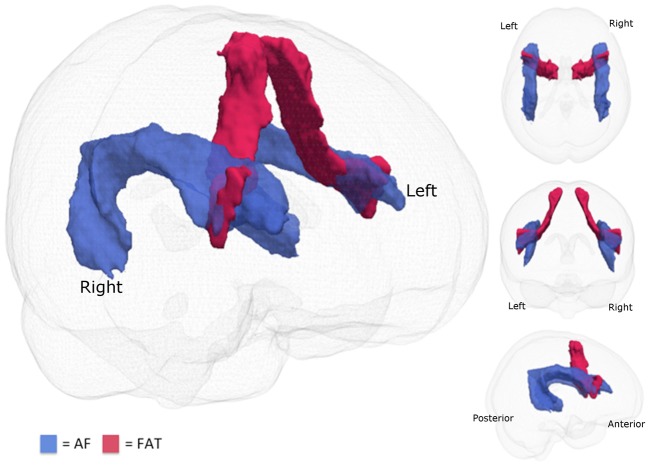
**Fiber tracts examined.** The two fiber tracts examined in this study are the arcuate fasciculus (AF; blue) and the frontal aslant tract (FAT; red).

The same preprocessing pipeline was used and applied BEDPOSTX (Behrens et al., 2003, 2007) to the 12 healthy adult subjects using MCMC sampling in order to obtain Bayesian estimates of the diffusion parameters to model crossing fibers for each voxel within the brain. Using PROBTRACKX (Behrens et al., 2007), connectivity distributions were generated using a curvature threshold of 0.2, a step length of 0.5 mm for 5000 streamline samples. For the tracing of the AF ROIs, waypoints and exclusion masks made according to the anatomical guidelines previously described in depth in Marchina et al. (2011) were used to identify the respective tracts in both hemispheres. The tractography of the FAT was carried out according to the descriptions in Catani et al. (2013) and Catani et al. (2016). Each corresponding set of 12 native reconstructed fiber tracts was thresholded at the 25th percentile of its intensity, normalized to the FMRIB standard FA template, binarized, and summed to craft a canonical tract, which was consequently used as a mask to extract mean FA values. Figure 1 shows the tracts that were examined in this study. Table 3 details the mean FA values for the 10 imaged AMMT participants in this study.

**Table 3:** **Participant imaging results at baseline**.

	Left arcuate fasciculus FA^1^	Right arcuate fasciculus FA	Left frontal aslant FA	Right frontal aslant FA
AMMT1	0.45	0.44	0.41	0.41
AMMT2	0.47	0.47	0.46	0.46
AMMT3	0.35	0.35	0.33	0.33
AMMT4	0.47	0.47	0.45	0.46
AMMT5	0.34	0.34	0.35	0.34
AMMT6	0.46	0.46	0.43	0.45
AMMT7	0.39	0.39	0.36	0.37
AMMT8	0.42	0.43	0.41	0.41
AMMT9	0.34	0.34	0.35	0.33
AMMT10	0.37	0.37	0.34	0.36
Mean (SD)	0.41 (0.05)	0.41 (0.05)	0.39 (0.05)	0.39 (0.05)

*^1^FA, Fractional anisotropy; a measure of fiber tract integrity and alignment*.

### Statistical Analyses

We wished to know which imaging variables (mean FA of right or left AF, mean FA of right or left FAT) significantly predicted change in fluency (as measured by change scores in our three speech production measures, syllable-initial consonants correct, response rate, and syllable insertions). In general, multiple regression analysis is most appropriate for this situation. However, regression techniques permit testing predictors of only one dependent variable at a time, thus increasing the number of tests performed and the likelihood of Type I errors. Thus, we employed canonical correlation analysis (CCA) as a way of assisting in the process of variable selection for the regressions; CCA allowed us to determine how and how much each of our measured variables contributed to the results. The second step, multiple regression, allowed us to construct a model that could determine whether specific independent variables were causally related to specific dependent variables.

CCA is a multivariate method used to assess the correlations between multiple predictor and outcome variables without elevating the Type I error rate (Sherry and Henson, 2005; Oslund, 2010). There are several advantages to using CCA, both on its own and to assist in variable selection for regression analyses. In addition to controlling for Type I errors, the multivariate nature of CCA makes it appropriate for research paradigms where there are multiple causes and multiple effects (Oslund, 2010). Here, the independent variables were FA for left and right AF and FAT; the dependent variables were the speech production measures.

**Figure 2:**
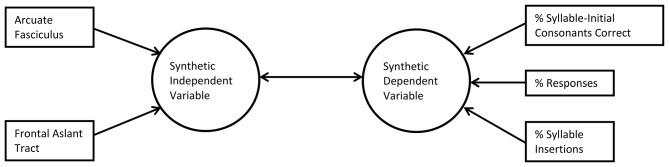
**Schematic of canonical correlation analysis (CCA; first root only).** In CCA, measured predictor variables (left) are related by structure coefficients (Pearson’s *r*) to a synthetic independent variable (lefthand arrows) and measured outcome variables (right) are related to a synthetic dependent variable (righthand arrows). The two synthetic variables are related by a canonical correlation coefficient, also a Pearson’s *r* (center double arrow).

CCA examines the relationship between multiple measured independent and dependent variables by creating synthetic (latent) independent and dependent variables or “roots”, which are linear combinations of the measured independent and dependent variables, respectively. In CCA there are as many roots as there are variables in the smaller of the two observed variable sets (here, three). The first root is constructed so as to maximize the Pearson correlation between the synthetic independent and dependent variables. Subsequent roots are constructed so as to maximize the Pearson *r* between the next pair of synthetic variables given the residual (unexplained) variance from the first root, and given that the second pair of synthetic variables is perfectly uncorrelated with the first. Because the synthetic variables are linear combinations of measured variables, and because each synthetic variable is uncorrelated with the others, CCA provides a way of understanding what factor the measured variables may have in common—similar to a principal components analysis. Furthermore, since the measured variables are combined to create the synthetic variables, and only the synthetic variables are compared to each other, the number of tests is minimized, which protects against Type I errors and avoids the need for a correction factor for multiple comparisons. In CCA, as in principal components analysis, only roots that explain a reasonable amount of the total variance are interpreted. Figure 2 shows a schematic diagram of CCA.

Using the results of the CCA to aid us in variable selection for the regression models, we then performed multiple regression analyses to understand whether FA values for the left and right AF or FAT significantly predicted the speech production measures that were significantly related to fluency, applying a Bonferroni correction to the final *p*-values from the regressions. All analyses were carried out in SPSS v. 23, using the syntax published in Oslund (2010) for the CCA analysis.

## Results

### Change Scores

Means, standard deviations, and ranges for the change scores for all 30 participants are reported in Table 2. Note that positive changes in % Syllable-Initial Consonants Correct and % Responses generally represent improvement in syllable-initial consonant production accuracy and increased ease of speech production, respectively. Change in % Syllable Insertions is more complex to interpret, but in general, a constant value near zero or a reduction in this figure is preferable. Note that while 7 of the 10 imaged AMMT children improved their % Syllable-Initial Consonants Correct scores, three did not. Nine of the 10 imaged AMMT children responded to more prompts at P25 compared to their Best Baseline (range: +1.7% to +31.1%). One imaged AMMT child responded to fewer prompts at P25 than at Best Baseline. For % Syllable Insertions, six imaged AMMT children had P25 scores less than 5% and change scores close to 5%. Two imaged AMMT children had syllable insertion rates above 10% at Baseline; one of these improved (decreased syllable insertion rates) at P25 and the other did not. Finally, two imaged AMMT children showed marked increases in their syllable insertion rates (from 13.3% to 21.4% and from 0% to 11.7% respectively).

### Canonical Correlation Analysis

The overall CCA model, with mean FA values for the left and right AF and the left and right FAT as independent variables and change scores for % Syllable-Initial Consonants Correct, % Responses, and % Syllable Insertions as dependent variables, was significant (Wilks’ *λ* = 0.002, *p* = 0.006). Dimension reduction analysis showed that the full model was significant, meaning that the cumulative effect of Root 1 to Root 3 was significant; that is, that all of the FA variables together explained a significant amount of the variance in the behavioral variables, *p* = 0.006. The cumulative effect of Root 2 to Root 3 and the effect of Root 3 alone were not significant. Thus, only the first root was interpreted.

Canonical correlation (Pearson’s *r*) between the synthetic independent and dependent variables for the first root was 0.998; and the squared correlation was 0.996, indicating that 99.6% of the variance in the dependents was explained by the independents. Table 4 reports the structure coefficients and the standardized canonical coefficients for the first root. Of the two measured independent variables, FA of the right AF and right FAT were the largest positive contributors to the fluency-related synthetic independent variable. FA of the left AF was also negatively related to the synthetic independent variable, as was FA of the left FAT (though only weakly). Of the measured dependent variables, change in % Syllable-Initial Consonants Correct was the largest contributor to the synthetic dependent variable and was positively related to it. Change in % Responses and % Syllable Insertions were negatively related to the synthetic dependent variable and contributed less to it.

**Table 4:** **Structure coefficients and standardized canonical coefficients for canonical correlation analysis (CCA; FA values by hemisphere)**.

	Variables	Standardized canonical coefficients	Structure coefficients
Independents	Left arcuate fasciculus FA	−1.776	0.965
	Right arcuate fasciculus FA	1.402	0.972
	Left frontal aslant tract FA	−0.623	0.918
	Right frontal aslant tract FA	1.960	0.981
Dependents	Change in % Syllable-Initial consonants correct	0.637	0.769
	Change in % Responses	−0.321	−0.324
	Change in % Syllable Insertions	−0.497	−0.817

### Regression Analysis

To test the hypotheses that FA of the AF significantly predicted change in % Syllable-Initial Consonants Correct and FA of the FAT significantly predicted change in % Responses and % Syllable Insertions, we tested a series of hierarchical regression models, entering predictor variables in the order of their hypothesized effect on the relevant outcome variable (informed by β-values from individual univariate regressions performed as part of SPSS’s CCA; uncorrected for multiple comparisons to avoid missing a potentially significant predictor).

Because four models were tested per regression analysis, we used a Bonferroni correction to establish appropriate α-values for each final model. For each regression, an α of 0.05/4, or 0.0125, was established as a correction factor for the *p*-values in each final model.

The model including FA of the left AF and right FAT as predictors of change in % Syllable-Initial Consonants Correct was significant before correction, *F*_(2,7)_ = 6.888, *p* = 0.022. However, the addition of predictor variables after the first resulted in only nonsignificant changes in *R*^2^. The model including only FA of the left AF significantly predicted change in % Syllable-Initial Consonants Correct even after correction, *F*_(1,8)_ = 15.704, *p* = 0.004; *R*^2^ = 0.663, *B* = 247.5.

The model including FA of both left and right AF and right and left FAT as predictors of % Responses was non-significant before correction, *F*_(4,5)_ = 0.920, *p* = 0.519. No variables significantly predicted change in % Responses.

Finally, the model including FA of the right and left FAT as predictors of change in % Syllable Insertions was significant before correction, *F*_(2,7)_ = 6.556, *p* = 0.025. However, the addition of predictor variables after the first again resulted in only nonsignificant changes in *R*^2^. The model including only FA of the right FAT significantly predicted change in % Syllable Insertions even after correction, *F*_(1,8)_ = 11.407, *p* = 0.01, *R*^2^ = 0.588, *B* = −95.6. Results for the significant hierarchical regression models are detailed in Table 5.

**Table 5:** **Summary of significant hierarchical regression analyses (FA values by hemisphere)**.

	*B*	*SE B*	β	*p*-value
*Variables predicting change in % Syllable-Initial consonants correct*
Left arcuate fasciculus FA	247.5	62.4	0.814	*p* = 0.004*
*Variables predicting change in % Syllable Insertions*
Right frontal aslant tract FA	−95.6	28.3	−0.767	*p* = 0.01*

*Both coefficients significant after Bonferroni correction, *p* < 0.0125.

## Discussion

In this study, we examined the relationship of fiber tract integrity for the AF and for the FAT to three variables indexing improvement in different aspects of fluent speech production in 10 MV children with ASD after 25 sessions of AMMT: change in % Syllable-Initial Consonants Correct, change in % Responses, and change in % Syllable Insertions. Several notable findings emerged that warrant further discussion.

First, we found that some of our participants experienced an increase in syllable insertion errors as they progressed through therapy, though most did not. To our knowledge, this is the first time this has been documented in the literature; however, Yoss and Darley (1974) do identify “addition” (insertion) errors as one of several variables distinguishing children with articulation disorders alone from those with articulation disorders and suspected childhood apraxia of speech. At this time, it is unclear how to interpret this finding: whether an increase in syllable insertion rate in some MV children is simply a developmental stage through which they will pass as they acquire spoken language or whether it is an integral part of the disorder that causes them to be MV in the first place (e.g., childhood apraxia of speech or an ASD-specific motor speech disorder) is unclear.

The second finding concerns the CCA, which investigated the relationship of multiple independent variables to multiple dependent variables by means of synthetic variables that account for the variance between two sets of measured variables. Results showed that the integrity of right-hemisphere tracts was positively correlated with the synthetic independent variable in this case, while integrity of the left-hemisphere tracts was negatively correlated with it. This suggests that integrity of the right-hemisphere FAT may contribute more to the synthetic fluency-related independent variable than does left-hemisphere tract integrity in MV children with ASD. This is consistent with work showing atypical lateralization of the AF (meaning that the typical leftward dominance in volume of the AF is altered) in MV children with ASD (Wan et al., 2012), in older verbal participants with ASD (Fletcher et al., 2010; Moseley et al., 2016), and with younger children with ASD (Fletcher et al., 2010; Joseph et al., 2014; Conti et al., 2016; Vogan et al., 2016).

The CCA analysis revealed that, as expected, increases in correct syllable-initial consonant production were positively related to the synthetic fluency-related dependent variable, while increases in syllable insertions were negatively related to it. Surprisingly, an increase in response rate was also negatively related to the fluency-related synthetic variable, though weakly. The weak relation of increases in response rate to the synthetic dependent variable may be due to the fact that changes in response rate were related to factors other than fluency, for example improvements in joint attention, compliance, or task comprehension.

The hierarchical regression analyses further elucidate the relationship of AF and FAT integrity with speech fluency. While the CCA showed that the imaging variables are related to speech accuracy and fluency to different extents, the regression analysis suggests that they are related to them in different ways. Specifically, left AF FA significantly predicted changes in % Syllable-Initial Consonants Correct after AMMT, and addition of other FA values did not significantly increase the variance explained. This was counter to our hypothesis that, in these children with altered AF laterality, integrity of the right AF would predict treatment response. The fact that left, but not right, AF FA values significantly predicted change in % Syllable-Initial Consonants Correct suggests that even in children with atypical lateralization of this tract, it is the integrity of the left hemisphere AF that may determine improvement in initial consonant production accuracy. Interestingly, initial analyses we performed using a laterality index for the AF and FAT ((FA of left tract − FA of right tract)/(FA of left tract + FA of right tract)) did not predict our outcome measures. Still, the current finding is consistent with the notion that the AF is involved in the mapping of articulatory actions and acoustic information to each other (Marchina et al., 2011). However, the AF may also subserve verbal memory functions that relate to speech production. The positive β-values for left AF FA in the regression analyses indicates that, as integrity of this tract increased, so did change in % Syllable-Initial Consonants Correct in the children in this study.

The careful reader will note that, while the β-value relating left AF FA to % Syllable-Initial Consonants Correct is positive, the standardized canonical coefficient relating it to the synthetic independent variable in the CCA analysis is negative. The nature of the synthetic variables in CCA analysis is not always straightforward to interpret, however; and the synthetic independent variable includes a component of laterality. Again, this result would be consistent with the finding of altered laterality of AF in participants with ASD (Fletcher et al., 2010; Wan et al., 2012; Joseph et al., 2014; Conti et al., 2016; Moseley et al., 2016; Vogan et al., 2016).

Another result was that the integrity of neither left nor right AF or FAT significantly predicted change in % Responses, supporting the notion that change in the number of prompts children responded to does not reflect improved fluency but is related to other factors. However, regardless of what was responsible for the increase in number of prompts our participants responded to, the fact that they were attempting more speech is a positive outcome. At the very least, it provided them with more opportunities to practice spoken language, an important improvement for this population.

Finally, right frontal aslant FA values significantly predicted change in % Syllable Insertions, and inclusion of additional FA variables did not significantly increase the variance explained. These results are consistent with the view that the FAT is implicated in the ability to retrieve and select motor plans for articulation during speech, though it does not necessarily play a role in establishing or monitoring correct auditory-motor mapping. The negative sign of β for right frontal aslant FA in the regressions indicates that, as integrity of this tract decreases, change in % Syllable Insertions increases, reflecting more syllable insertion errors (more “extra” syllables produced).

The finding that FA of the right-hemisphere FAT predicted change in % Syllable Insertions is surprising, given the general thinking that it is the left FAT that is implicated in the ability to initiate fluent speech (Kronfeld-Duenias et al., 2016). Two aspects of this finding are important. First of all, the measure of fluency used in Kronfeld-Duenias et al. (2016) was speech rate (syllables/second), while our measure of fluency concerned extraneous syllable production—two somewhat opposite types of dysfluency. The authors did not report the correlation of FA of the FAT with the other speech-related variables they measured, percent stuttered syllables, percent stuttering-like dysfluencies, or stuttering severity score. Thus, it may be the case that while impaired integrity of the left FAT relates to speech stoppage, impaired integrity of the right FAT relates to a high degree of syllable insertions. Alternatively, since FA reflects not only myelination of a fiber tract but also fiber density and axonal diameter, it may be that different diffusivity components are associated with speech arrest than with syllable-insertion errors. Future work investigating axial, radial, and mean diffusivity and their relationship with specific types of dysfluency is needed to answer these questions.

The second important aspect of our results is that they are consistent with the findings of Broce et al. (2015). These authors found evidence of right laterality of the FAT in children between the ages of 5 and 8, an age range similar to that of the participants in our study. They speculate that this tract functions as an “action selection loop” for speech and that there may be a stronger relationship between white-matter development in the FAT and measures of speech performance in children with speech-sound disorders or language impairment. Certainly, an impairment in the process of selecting motor actions for speech articulation could result in difficulty inhibiting the selection of unwanted motor actions and result in a high rate of “extra syllable” production. As mentioned above, it is difficult to know how to fit the increase in syllable-insertion errors seen in some of our participants into adult models of dysfluency. Anecdotally, we observed little evidence of muscular tension or struggle in the speech of the children who produced the most syllable insertions. This lends credence to the idea that the syllable insertions produced by our participants were not the stuttering-like dysfluencies produced by people who stutter (Yairi, 2007) but may have a different origin.

### Clinical Implications

The current results can inform our clinical practice in that they may indicate the presence of a biomarker that can be used to predict treatment response from AMMT. Though some of the imaged participants showed significant improvement after just 10 sessions of AMMT (and smaller improvement thereafter), as mentioned, other participants did not show an improvement after 25 sessions of AMMT. Yet, given the limited developmental time available to these children, and limited funds available to their families, identification of a reliable predictor of treatment response would make an important positive difference in routing these children to an appropriate therapy or communication modality.

However, many questions still remain to be investigated. For example, it will be important to compare outcome measures to both pre- and post-treatment imaging to identify changes in diffusion parameters as a result of treatment. The training and desensitization necessary to scan MV children with autism more than once are considerable, and the loss of data when children are unable to participate is costly. Thus, carefully-designed training protocols that move at a child’s own speed are needed to successfully image this population.

In terms of DTI parameters other than FA, we would expect to find changes in both radial and axial diffusivity, since with behavioral improvement, the connectivity between temporal and frontal lobes might increase and therefore myelination might change. In addition, it is possible that axonal sprouting at the nodal points of the AF could occur. We have documented such axonal sprouting in an intense treatment study (Wan et al., 2014) and hypothesize that it could appear as change in axial diffusivity.

Finally, as mentioned, the increases in syllable insertions that some children experienced during the study must be better understood, as must the roles of the left and right hemisphere in producing accurate, fluent speech without unusual numbers of syllable insertions. Longitudinal studies tracking speech development in MV children are needed to understand the nature and prevalence of this increase. Similarly, the factors contributing to the increase in response rate should be investigated in greater detail, as it is highly likely that they play a role in why MV children remain MV. Furthermore, we do not yet understand how the combination of factors such as social impairment, severity of repetitive behaviors, language disorders, cognitive impairment and motor impairment individually or collectively produce the range of spoken-language behaviors we see in MV children with ASD.

### Limitations of the Present Study

A chief limitation of this study is the small number of participants. It is quite challenging for MV children with ASD to participate in imaging studies without sedation, but not impossible. Given adequate behavioral support and the possibility of scanning when the children are naturally asleep, larger cohorts of children in this population might be successfully imaged and those findings related to variables of clinical interest. Another, related, consideration is the fact that the large majority of our participants were male. Given recent evidence that ASD may manifest differently in males and females, future work should aim to include a larger proportion of girls. Advances in movement correction technology will aid in the interpretation of imaging findings as well. Furthermore, the lack of a large-enough group of typically-developing children whose images could be used to construct canonical tracts for the AF and FAT necessitated the inclusion of tracts from healthy adults so that regional FA values and laterality differences could be obtained. Nevertheless, the anatomical course of the tracts in the healthy adults, the small group of typically developing children, and the larger group of autistic children were carefully evaluated, and the canonical tracts of adults were found to be representative of the typical tracts seen in children. A final limitation of this study is the small number of perceptually-based speech measures that were investigated. While measures such as percent consonants or vowels correct and rates of different error types are ecologically valid and traditionally used in phonological analyses of developing speech, they are subject to bias and are time-consuming to analyze. Future work should aim to include acoustic and kinematic analyses of the speech of MV children with ASD, as these objective measures will only add to our understanding of the strengths and challenges these children bring to the task of learning spoken language.

## Author Contributions

GS, KC, JK and AN: substantial contributions to the conception or design of the work; or the acquisition, analysis, or interpretation of data for the work; drafting the work or revising it critically for important intellectual content; final approval of the version to be published; agreement to be accountable for all aspects of the work in ensuring that questions related to the accuracy or integrity of any part of the work are appropriately investigated and resolved.

## Funding

Funding for this study was provided by the Nancy Lurie Marks Family Foundation, Autism Speaks, and by National Institutes of Health (NIH) P50-DC 13027.

## Conflict of Interest Statement

The authors declare that the research was conducted in the absence of any commercial or financial relationships that could be construed as a potential conflict of interest.

## Footnotes

^1^www.fmrib.ox.ac.uk/fsl
